# Targeted visual cortex stimulation (TVCS): a novel neuro-navigated repetitive transcranial magnetic stimulation mode for improving cognitive function in bipolar disorder

**DOI:** 10.1038/s41398-023-02498-z

**Published:** 2023-06-08

**Authors:** Dandan Wang, Lili Tang, Caixi Xi, Dan Luo, Yin Liang, Qi Huang, Zhong Wang, Jingkai Chen, Xudong Zhao, Hetong Zhou, Fei Wang, Shaohua Hu

**Affiliations:** 1 Department of Psychiatry, the First Affiliated Hospital, Zhejiang University School of Medicine, Hangzhou, 310003 China; 2 The Key Laboratory of Mental Disorder’s Management in Zhejiang Province, Hangzhou, 310003 China; 3 Early Intervention Unit, Department of Psychiatry, Affiliated Nanjing Brain Hospital, Nanjing Medical University, Nanjing, 210000 P.R. China; 4 Functional Brain Imaging Institute of Nanjing Medical University, Nanjing, 210000 P.R. China; 5 Ward Five of The Third People’s Hospital of Jiashan County, Jiaxing, 314000 China; 6 Taizhou Second People’s Hospital, Taizhou, 318000 China; 7 Nanchong Psychosomatic Hospital, Nanchong, 637000 China; 8 Huzhou Third municipal hospital, Huzhou, 313000 China; 9 Brain Research Institute of Zhejiang University, Hangzhou, 310003 China; 10 Zhejiang Engineering Center for Mathematical Mental Health, Hangzhou, 310003 China; 11 MOE Frontier Science Center for Brain Science & Brain-Machine Integration, Zhejiang University, Hangzhou, 310003 China

**Keywords:** Bipolar disorder, Physiology

## Abstract

A more effective and better-tolerated site for repetitive transcranial magnetic stimulation (rTMS) for treating cognitive dysfunction in patients with bipolar disorder (BD) is needed. The primary visual cortex (V1) may represent a suitable site. To investigate the use of the V1, which is functionally linked to the dorsolateral prefrontal cortex (DLPFC) and anterior cingulate cortex (ACC), as a potential site for improving cognitive function in BD. Seed-based functional connectivity (FC) analysis was used to locate targets in the V1 that had significant FC with the DLPFC and ACC. Subjects were randomly assigned to 4 groups, namely, the DLPFC active-sham rTMS (A1), DLPFC sham-active rTMS (A2), ACC active-sham rTMS (B1), and ACC sham-active rTMS groups (B2). The intervention included the rTMS treatment once daily, with five treatments a week for four weeks. The A1 and B1 groups received 10 days of active rTMS treatment followed by 10 days of sham rTMS treatment. The A2 and B2 groups received the opposite. The primary outcomes were changes in the scores of five tests in the THINC-integrated tool (THINC-it) at week 2 (W2) and week 4 (W4). The secondary outcomes were changes in the FC between the DLPFC/ACC and the whole brain at W2 and W4. Of the original 93 patients with BD recruited, 86 were finally included, and 73 finished the trial. Significant interactions between time and intervention type (Active/Sham) were observed in the scores of the accuracy of the Symbol Check in the THINC-it tests at baseline (W0) and W2 in groups B1 and B2 (*F* = 4.736, *p* = 0.037) using a repeated-measures analysis of covariance approach. Group B1 scored higher in the accuracy of Symbol Check at W2 compared with W0 (*p* < 0.001), while the scores of group B2 did not differ significantly between W0 and W2. No significant interactions between time and intervention mode were seen between groups A1 and A2, nor was any within-group significance of FC between DLPFC/ACC and the whole brain observed between baseline (W0) and W2/W4 in any group. One participant in group B1 experienced disease progression after 10 active and 2 sham rTMS sessions. The present study demonstrated that V1, functionally correlated with ACC, is a potentially effective rTMS stimulation target for improving neurocognitive function in BD patients. Further investigation using larger samples is required to confirm the clinical efficacy of TVCS.

## Introduction

Bipolar disorder (BD) is a chronic disorder with recurrent periods of depression and mania or hypomania. It has a lifetime prevalence of 2.4% for the bipolar disorder spectrum and affects over 1% of the global population [1]. There is increasing evidence that BD is associated with cognitive impairment, even during euthymic periods [2, 3]. These persistent cognitive impairments are considered to be independent of mood symptoms and lead to disability in both occupational and interpersonal functioning in BD patients [4, 5], thus reducing their quality of life.

Thus, it is useful to focus not only on improvement in the emotional symptoms of BD in intervention studies, but also on the preservation and enhancement of cognitive function. Evidence during the past few decades has shown that noninvasive techniques of brain stimulation, such as repetitive transcranial magnetic stimulation (rTMS), have positive effects on improving cognitive function. A paper published in *Science* reported that a targeted TMS pulse results in the brief generation of an active item in working memory, suggesting that this stimulation may enhance brain activity and thus improve memory [6]. Moreover, targeting of the left dorsolateral prefrontal cortex (DLPFC) by high-frequency (10 Hz) rTMS was found to improve the recognition of facial expression in schizophrenia [7]. Studies have demonstrated that high-frequency rTMS over the left DLPFC improved cognitive impairment in bipolar depression [8, 9], and a double-blind, randomized, sham-controlled trial observed improvements in cognition in euthymic patients after 10 consecutive days of high-frequency rTMS treatment of the left DLPFC [10]. However, there has been no final conclusion on the effect of rTMS targeting the left DLPFC on the mitigation of cognitive impairment. The therapeutic effects of rTMS over the left DLPFC were reported to be relatively modest for cognitive enhancement in unipolar depression [11] and a meta-analysis even showed no significant improvement in cognitive function in patients with various neuropsychiatric disorders, including unipolar or bipolar depression, panic disorder (PD), posttraumatic stress disorder (PTSD), and obsessive-compulsive disorder (OCD) [12]. In schizophrenia patients, it was found that high-frequency rTMS targeting the left DLPFC was no better than sham rTMS for mitigating cognitive impairment [13]. These results suggest that the left DLPFC may not represent the optimal rTMS target for treating cognitive impairment.

The effects of rTMS on improving cognitive function in BD patients appear to be significantly affected by its localization and target of stimulation. Further investigation is, therefore, required to identify key treatments and potential stimulation targets other than the left DLPFC.

As is well-known, the primary visual cortex (V1) plays a vital role in the transmission of visual information transmission and receives input from the retina through the lateral geniculate nucleus (LGN) in the thalamus, processing the information by the extraction of basic features from the visual world [14]. Notably, neurons in the early-visual cortex (VC) (V1–V4) not only process incoming visual information but also contribute to higher-level cognitive processing, specifically, working memory, decision-making, attention, and imagery. Hence, the early VC (V1–V4) can be considered a cognitive blackboard for both reading and writing by higher-level visual areas, allowing the efficient exchange of information [15]. In addition, V1 plays a role in iconic memory. Iconic memory refers to the brief, high-capacity memory storage in visual perception, which is a brief, detailed representation of a brief visual image [16]. Studies suggest that iconic memory is primarily associated with the decay of V1 activity following brief visual stimuli [17]. V1 can not only be activated by incoming visual information, but can also participate in visual working memory even in the absence of visual information input [18]. Visual working memory is supported by neural feedback from higher-order brain areas, and the agranular layer of V1 is the primary target for receiving this neural feedback and is involved in maintaining specific visual features (i.e., orientation) during working memory [19]. Previous studies have also indicated that a larger V1 volume is predictive of greater storage of visual working memory [20]. V1 is also engaged in an individual’s attention processes. The theory of visual attention proposes the existence of a visual saliency map, which guides individuals to notice the most obvious stimuli in complex scenes, and V1 is involved in the encoding of the saliency map [21]. Furthermore, TMS has been shown to induce significant remodeling of essentially mature structures in the early VC in a noninvasive manner [22]. Lines of evidence suggest a close relationship between emotional symptoms in depression and the alterations of the visual cortex structure and function, and antidepressants can make changes to the electrophysiological characteristics and neurotransmitters in the visual cortex [23]. More recently, neuro-navigated rTMS was applied to the V1 area in patients with major depressive disorder (MDD), with good tolerance and results [24]. Together, these findings affirm the important role of V1 in the regulation of depressive symptoms and cognitive function, and indicate the potential of the V1 area as a target for mitigating cognitive impairment associated with psychiatric disorders.

However, the neural anatomy of the V1 varies significantly between individuals, with three to fourfold variations in the surface area between individuals [25]. Precise positioning and targeting of V1 are essential to ensure the efficacy of rTMS. It is well-known that the DLPFC forms a major part of the central executive network [26], with links to both the default mode network (DMN) [27] and the sensorimotor network (SMN) [28] and playing a vital role in cognitive control and emotional processing in BD. The anterior cingulate cortex (ACC) is thought to monitor conflict or error during response selection, and is involved in the modulation of cognitive control over motivation-driven behavior in BD [29] as well as being involved in cognitive control networks. Because of the vital functions of both the ACC and DLPFC in cognitive processes, the current study has defined two areas in V1 as targets due to their significant links with the DLPFC and ACC. Thus, to investigate whether stimulation of the V1 in areas functionally associated with the ACC and DLPFC is effective for improving neurocognitive function in BD patients, we set up and conducted a sham-controlled randomized double-blind trial.

## Methods

### Trial design

**Fig. 1:**
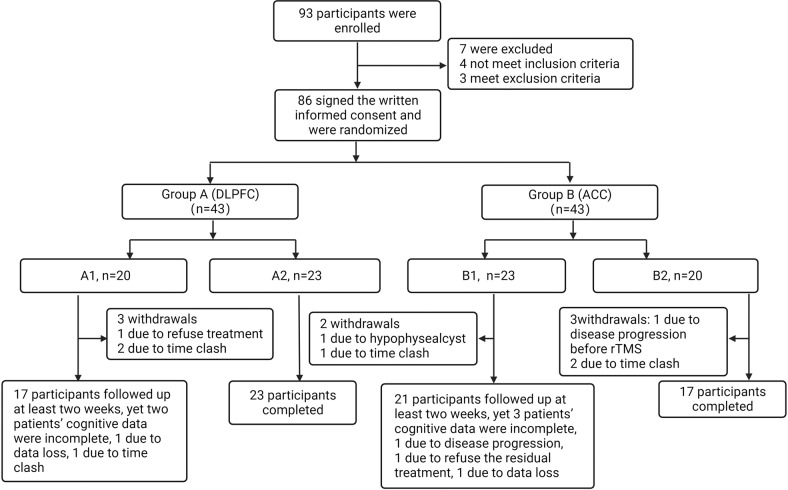
Flow chart of study recruitment and the reasons for participant withdrawal. DLPFC dorsolateral prefrontal cortex, ACC anterior cingulate cortex, A1, DLPFC active-sham rTMS Group, A2, DLPFC sham-active rTMS Group, B1, ACC active-sham rTMS Group, B2, ACC sham-active rTMS Group.

The study was conducted at the First Affiliated Hospital, Zhejiang University School of Medicine, Hangzhou, China, from March 1, 2020, to June 30, 2021. The study was a double-blind randomized controlled trial. The participants were allocated to one of two groups, A and B. In group A, the V1 areas that were functionally associated with the DLPFC were targeted, while participants in group B were treated by targeting the V1 area functionally linked with the ACC. In addition, both groups were then randomly divided into two subgroups, namely, A1/A2 and B1/B2, according to the stimulating pattern; the allocation was performed using a random number table generated by a computer by trained staff. All the participants were treated with rTMS once daily, five times per week for four weeks. Specifically, groups A1 and B1 received 10 days of active treatment followed by 10 days of sham treatment, referred to as DLPFC/ACC active-sham rTMS. In contrast, groups A2 and B2 received 10 days of sham treatment followed by 10 days of active treatment, referred to as DLPFC/ACC sham-active rTMS (see Fig. 1). All participants maintained their primary medication throughout the trial. Both the participants and the investigators who evaluated cognitive function and emotional symptoms were fully blinded to the allocation of the participants. All procedures involving human subjects/patients in the study were approved by the Clinical Research Ethics Committee of the First Affiliated Hospital, Zhejiang University School of Medicine, and complied with the ethical standards of the relevant national and institutional committees on human experimentation and with the Helsinki Declaration of 1975, as revised in 2008. Written informed consent was received from each participant before participation in the study. The study has been registered with the Chinese Clinical Trial Registry (http://www.chictr.org.cn/enIndex.aspx), with the registration number ChiCTR2000030675.

### Sample size

The optimum size of the sample was determined using the difference in means between the baseline and post-treatment Symbol Check accuracy scores, which were 0.26 in group A1, 0.08 in group A2, 0.2 in group B1, and 0.05 in group B2, according to the preliminary experiment. Furthermore, it was also calculated based on the differences in the standard deviations of Symbol Check accuracy scores, which were 0.27 in group A1, 0.28 in group A2, 0.29 in group B1, and 0.29 in group B2, according to the preliminary experiment. The overall number of participants was calculated using power analysis in PASS 15.0. This showed that for 80% power at a two-tailed level of significance of 0.05, each group should contain 37 participants for the four-week treatment period. This included an estimated withdrawal rate of 10%. Thus, a minimum of 148 participants (1:1:1:1 randomization ratio) was required.

### Participants

Outpatients with BD at the Department of Psychiatry, First Affiliated Hospital, Zhejiang University School of Medicine, were recruited by advertisement from March 1, 2020, to June 30, 2021. The inclusion criteria included: (i) 16–65 years of age; (ii) diagnosis of BD established by two professional psychiatrists, in accordance with the Mini-International Neuropsychiatric Interview (MINI); (iii) stable medication; (iv) more than three months of clinical remission assessed by a score ≤6 on the Young Mania Rating Scale (YMRS) [30] and a score ≤7 on the 17-item Hamilton Depression Rating Scale (HDRS-17) [31] before group assignment; (v) self-reported cognitive impairments with perceived deficits questionnaire-depression (PDQ-D) ≥17 [32], (vi) right-handedness, (vii) education ≥9 years. The exclusion criteria included: (i) comorbidities of any other mental disorder in the Diagnostic and Statistical Manual of Mental Disorders, Fifth Edition (DSM-5); (ii) history of serious neurologic illnesses, such as epilepsy or traumatic brain injury; (iii) significant and unstable medical conditions, including diabetes or cardiovascular, hematological, endocrine, liver, or kidney disease; (iv) history of substance or alcohol abuse; (v) pregnant or breast-feeding women; (vi) achromatopsia, hypochromatopsia, or dysaudia; (vii) medication of antidepressant anticholinergic agents; (viii) contraindications for subjecting to MRI scan.

### Assessments

The PDQ-D was used to screen the cognitive impairment in the participants at baseline. The HDRS-17 and YMRS were used for assessing symptom severity at different time points. The researcher evaluators were trained for assessment consistency. The occurrence of adverse events was documented during the treatment process. The THINC-integrated tool (THINC-it) used was a self-operated iPad version that has been shown to be both reliable and valid for evaluating the cognitive impairments of BD depression [33]. The THINC-it consists of Spotter, Symbol Check, Codebreaker, Trails, and the Perceived Deficits Questionnaire for Depression-5-item (PDQ-5-D), corresponding to another five conventional neuropsychological tests, namely, the Identification Task (IDN), One-Back Task (1-back), Digit Symbol Substitution Test (DSST), Trail Making Test-Part B (TMT-B), and Perceived Deficits Questionnaire for Depression (PDQ) [34] (see Supplementary Table 1), to assess attention, speed of processing, working memory, and cognitive and executive function [35]. The cognitive function of all the participants was evaluated by THINC-it at baseline (W0), week 2 (W2), and week 4 (W4). The primary outcomes were changes in the scores of the five tests in THINC-it at W2 and W4. The secondary outcomes were changes in the functional connectivity (FC) between DLPFC/ACC and the whole brain at W2 and W4.

### Neuroimaging method and analysis

#### Imaging acquisition

Imaging was performed on a 3.0 Telsa scanner (GE SIGNA) with a standard whole-head coil at the First Affiliated Hospital, Zhejiang University School of Medicine. The participants were asked to lie on the scanner with their eyes closed. Foam cushions were positioned on either side of the head to restrict head motion, and earplugs were used to reduce the noise. High-resolution 3D anatomical images were captured through T1-weighted magnetization prepared rapid acquisition gradient-echo (MPRAGE) sequence. The parameters used were: TR = 7.1 ms; TE = 2.9 ms; FOV = 260 × 260 mm^2^; matrix size = 256 × 256; slices = 146; slice thickness = 1 mm; flip angle = 8°. Functional images were acquired by the echo planar imaging (EPI) sequence, with the following parameters: TR = 1800 ms; TE = 30 ms; FOV = 240 × 240 mm^2^, gap = 0.8 mm; matrix size = 64 × 64; slices = 28; slice thickness = 4 mm; time points = 180.

#### Data preprocessing

Preprocessing of the fMRI data was done on the DAPBI platform (http://rfmri.org/dpabi). This involved DICOM conversion and the removal of the initial ten-time points to guarantee signal stability. Slice 27 was used as a reference for the timing of slices. Head motions were realigned, and participants showing head motions of over 1.5 mm in any direction (x, y, or z) or over 1.5° were excluded. The T1 and functional images were manually reoriented to the anterior commissure, with T1 co-registered to the functional images, the segment co-registered T1 images by Dartel, and the T1 images registered to a standardized Montreal Neurologic Institute (MNI) space brain. The transformation relation was applied to the functional images and 24 head-motion covariates (Friston24) were removed. Resampling of the images to a spatial resolution of 3 × 3 × 3 mm was performed and spatial smoothing was done with a Gaussian kernel (FWHM = 4 mm). Signals from the cerebrospinal fluid and white matter were removed using regression analysis, the images were filtered (0.01–0.1 Hz), and the motion was scrubbed.

#### Stimulating target and rTMS parameters

Several regions of interest (ROIs) were defined in DLPFC and ACC according to previously reported coordinates from earlier studies on BD (−32, 42, 32) (−4, 50, 4) [36, 37]. The DLPFC and ACC seeds were identified by 9-mm spheres centered on the coordinates (−32, 42, 32) (−4, 50, 4) and these two seeds were used to construct the FC maps. The voxel average in each ROI was used to assess the time course. The above coordinates were used to identify the optimized TMS targeting coordinates in the V1 using the computed seed-based FC with the a priori identified ROIs in the DLPFC and ACC of 30 healthy subjects. The V1 stimulation sites were first determined according to their voxel-wise FC with the previously defined ROIs in the DLPFC and ACC. The V1 sites were significantly correlated with the DLPFC and ACC (voxel-wise *p* < 0.001, cluster-wise FWE-corrected *p* < 0.05). The DLPFC was significantly anti-correlated with V1 (6, −63, 15) with *r* = −0.179, while the ACC was significantly correlated with V1 (−3, −66, 18) with *r* = 0.269. These two coordinates in V1 were then chosen as the rTMS targets of group A and group B, respectively (see Supplementary Fig. 1).

After positioning the TMS coil over the V1 coordinates calculated from the individual MRI images, a 3D curvilinear reconstruction of the brain was generated (Brainsight TMS navigation system).

The MRI-based neuro-navigation targeting and rTMS were performed by trained staff using Magstim Rapid2 rTMS devices (The Magstim Company, Whitland, UK) with a figure-of-eight coil. Each study participant received 20 days of rTMS treatment. The participants in groups A1 and B1 were treated with 10 days of active rTMS followed by 10 days of sham rTMS treatment, while, in contrast, groups A2 and B2 underwent 10 days of sham rTMS treatment before 10 days of active rTMS. Each daily rTMS treatment consisted of 60 five-second 10-Hz trains delivered at 110% of the resting motor threshold with inter-train intervals of 20 s (i.e., 3000 pulses per session). For the sham treatment, the coil was directed to the same target with the simulation of scalp sensations with the production of the same sounds, without the application of the magnetic field.

### Statistical analysis

All data were analyzed using SPSS version 24.0 (IBM Corp., Armonk, NY, USA). The data are expressed as mean ± standard deviation (SD). To compare the general demographic data, one-way ANOVA was used for comparing means between the four groups, and nonparametric data were compared using the Chi-square test.

Repeated-measure of covariance was used for most of the statistical analysis. The scores of THINC-it at W0, W2, and W4 were evaluated as the outcome variables. We conducted four types of comparisons to analyze the effects of the rTMS intervention mode (active/sham) and the stimulating mode (ACC/DLPFC) on cognitive function. The first comparison included all participants with the targeting of the ACC; the between-participant factor was set as the group (active/sham), and the within-participant factor was set as the time point (W0/W2) and (W2/W4), respectively, to examine the interaction between time and intervention mode. The second comparison included all participants with the DLPFC target; the between-participant and within-participant factors were the same as those used for the ACC target. The third comparison included all participants who received active-sham stimulation; the between-participant factor was the group (ACC/DLPFC), and the within-participant factor was the time point (W0/W2) and (W2/W4). For the last comparison, we included all participants who received sham-active stimulation, using the same between-participant and within-participant factors as the active-sham stimulation. Significance was assessed by the interaction between the time point and the mode of stimulation, indicating whether or not the change in outcome variables over time differed between the mode of stimulation.

The mood-symptom data were analyzed using repeated-measures ANOVA, with the treatment group (A1, DLPFC active-sham rTMS Group; A2, DLPFC sham-active rTMS Group; B1, ACC active-sham rTMS Group; B2, ACC sham-active rTMS Group) as the between-participant factor and time point when cognitive and clinical measurements were conducted (W0, W2, W4) as the within-participant factor. The threshold for significance level was defined as *P* < 0.05.

The FC between the voxels in the whole brain and two ROIs, DLPFC and ACC, was determined separately by Pearson’s correlation coefficients in DPABI (http://rfmri.org/dpabi). This was followed by the transformation of the correlation coefficients from r- to z-values using the Fisher-z transformation. Voxel-wise differences between the groups were analyzed by one-way ANOVA in SPM8 (http://www.fil.ion.ucl.ac.uk/spm) and group effects were analyzed with one-sample t-tests (FDR corrected *P* < 0.05). The images of the voxel-wise seed-based FC maps were adjusted using multiple comparisons (voxel-wise P < 0.001, cluster level FWE-corrected *P* < 0.05), and paired t-tests were used to determine differences in the whole-brain FC network and the DLPFC/ACC seeds between W0 and W2/W4.

## Results

### Participant demographics

Out of 93 participants, seven were not allocated to groups as they did not meet the inclusion criteria or did meet the exclusion criteria. Eighty-six qualified remitted BD patients were randomized and 73 participants completed all the treatments, including 15 participants in group A1, 23 in group A2, 18 in group B1, and 17 in group B2, while the remaining five participants completed at least 2 weeks’ follow-ups (see Fig. 1). The baseline demographic information on the participants and their scores on the clinical scales are summarized in Table 1. There were no significant differences between the groups in age, sex, education years, disease course, body mass index, YMRS score, HDRS-17 score, or PDQ-5-D score (*p* > 0.05).

**Table 1:** Demographic information and clinical scale scores of all participants at baseline.

	DLPFC		ACC		Analysis *F*/*χ*^*2*^	*p* value
	A1 (*n* = 15)	A2 (*n* = 23)	B1 (*n* = 18)	B2 (*n* = 17)		
Age	22.47 ± 11.13	24.91 ± 11.01	20.92 ± 5.97	21.35 (5.11)	0.851	0.471
Sex (Male/Female)	6/9	8/15	8/10	4/13	1.828	0.609^*^
Education years	12.33 ± 2.67	12.98 ± 2.49	12.78 ± 2.92	12.21 ± 2.33	0.372	0.774
Disease course (Months)	47.67 ± 58.45	59.39 ± 93.39	29.89 ± 21.51	54.17 ± 30.05	0.836	0.479
Body mass index	21.89 ± 5.15	23.28 ± 3.80	21.61 ± 4.52	23.72 ± 4.72	0.931	0.431
YMRS score	1.13 ± 1.64	1.43 ± 2.25	2.06 ± 2.51	2.00 ± 1.97	0.730	0.537
HDRS-17 score	4.40 ± 2.32	3.78 ± 2.54	4.11 ± 2.52	5.59 ± 1.62	2.145	0.102
PDQ-5-D	33.93 ± 15.90	29.39 ± 10.96	33.78 ± 16.71	31.71 ± 12.22	0.467	0.706

*DLPFC* dorsolateral prefrontal cortex, *ACC* anterior cingulate cortex, *YMRS* Young manic rating scale, *HDRS-17*, Hamilton depression rating scale; *PDQ-5-D* perceived deficits questionnaire for depression-5-item, *A1, DLPFC* active-sham rTMS Group, *A2, DLPFC* sham-active rTMS Group, *B1, ACC* active-sham rTMS Group, *B2, ACC* sham-active rTMS Group.

*p* value: * indicates the chi-square test result.

### Mood symptoms

There were no significant differences observed in the YMRS and HDRS-17 scores at the various time points (see Supplementary Table 2).

### Cognitive function

**Fig. 2:**
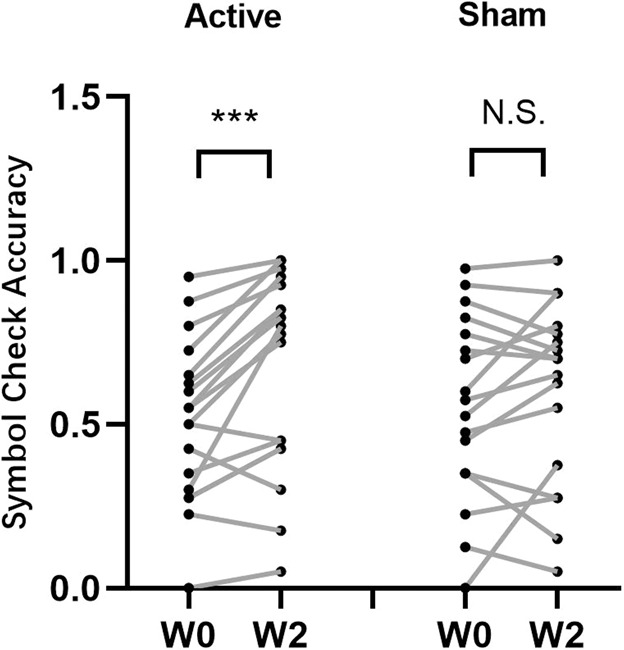
Pairwise comparisons of the symbol check accuracy scores between W0 vs. W2 for the active and sham groups in participants with ACC targeting. Each data point represents participants with recorded symbol check accuracy scores at that time point. ACC anterior cingulate cortex, W0 week 0, W2 week 2; ***P < 0.001; NS non-significant.

In terms of the effects of interaction between the time of assessment and the intervention mode (active/sham) in group B (ACC active-sham/ sham-active rTMS Group), a significant interaction effect was observed in the scores of the Symbol Check Accuracy measurement between W0 and W2 (see Table 2), while no significant interactions between group and time were found for the other five scores when comparing W0 and W2, nor in any of the six scores when comparing W2 and W4 (see Table 2). A significant interaction was observed for the ACC target, with paired t-test analysis showing that the participants who received active stimulation had higher Symbol Check Accuracy scores (at W2) compared to those at baseline (W0). However, no significant difference was found for participants who received sham stimulation (see Fig. 2). In the second comparison to investigate the effect of interaction between time point and intervention mode using the six THINC-it cognitive function scores in participants with the targeting of the DLPFC, there was no significant interaction, shown both when comparing the six scores between W0 and W2 and between W2 and W4 (see Table 3). The third comparison exploring the interaction effect between time and stimulation target (ACC/DLPFC) in participants with active-sham stimulation, showed no significant effects on the scores of the six tests between W0 and W2 nor between W2 and W4 (see Supplementary Table 3). Similar results were obtained in the final comparison of participants who received sham-active stimulation, where no significant time-by-group (ACC/DLPFC) interactions were found in the six scores between W0 and W2 and between W2 and W4 (see Supplementary Table 3).

**Table 2:** Interaction between the time of measurement and group (active/sham) on six THINC-it cognitive function scores in participants with ACC targeting.

	ACC active-sham rTMS Group			ACC sham-active rTMS Group			*P*1	P2
	W0	W2	W4	W0	W2	W4		
	(*N* = 18)			(*N* = 17)				
Spotter CRT	−0.26 ± 0.13	−0.30 ± 0.11	−0.31 ± 0.1	−0.20 ± 0.15	−0.29 ± 0.08	−0.31 ± 0.08	>0.05	>0.05
Symbol Check (Time)	0.03 ± 0.11	−0.04 ± 0.09	−0.08 ± 0.08	0.06 ± 0.11	−0.03 ± 0.07	−0.07 ± 0.08	>0.05	>0.05
Symbol Check (Accuracy)	0.51 ± 0.25	0.67 ± 0.30	0.71 ± 0.28	0.56 ± 0.29	0.60 ± 0.28	0.75 ± 0.24	0.037*	>0.05
Codebreaker DSST	48.44 ± 16.23	57.00 ± 23.66	59.72 ± 21.68	54.88 ± 17.92	61.76 ± 16.63	65.94 ± 12.66	>0.05	>0.05
Trails	77.51 ± .200.46	38.28 ± 59.06	34.11 ± 64.99	29.63 ± 20.54	22.12 ± 11.21	19.17 ± 10.13	>0.05	>0.05
PDQ-5-D	11.17 ± 3.78	8.78 ± 3.19	7.33 ± 2.99	10.76 ± 5.23	9.88 ± 4.85	8.94 ± 4.42	>0.05	>0.05

*ACC* anterior cingulate cortex, *rTMS* repetitive transcranial magnetic stimulation, *W0* week 0, *W2* week 2, *W4* week 4, *P1* the interaction between time of measurement (W0/W2) and group(active/sham), *P2* the interaction between time of measurement (W2/W4) and group(active/sham).

*The statistical threshold is *p* < 0.05.

**Table 3:** Interaction between the time of measurement and group (active/sham) on six THINC-it cognitive function scores in participants with DLPFC targeting.

	DLPFC active-sham rTMS Group			DLPFC sham-active rTMS Group			*P*1	*P*2
	W0	W2	W4	W0	W2	W4		
	(*N* = 15)			(*N* = 23)				
Spotter CRT	−0.20 ± 0.15	−0.27 ± 0.12	−0.30 ± 0.11	−0.22 ± 0.13	−0.25 ± 0.11	−0.26 ± 0.11	>0.05	>0.05
Symbol Check (Time)	0.01 ± 0.11	−0.02 ± 0.09	−0.05 ± 0.10	0.05 ± 0.10	0.02 ± 0.09	−0.04 ± 0.08	>0.05	>0.05
Symbol Check (Accuracy)	0.54 ± 0.31	0.71 ± 0.26	0.74 ± 0.29	0.43 ± 0.27	0.53 ± 0.29	0.66 ± 0.26	>0.05	>0.05
Codebreaker	49.80 ± 17.17	56.80 ± 21.51	59.67 ± 21.75	42.91 ± 16.58	53.04 ± 12.26	56.57 ± 14.73	>0.05	>0.05
Trails	27.50 ± 14.52	22.65 ± 9.00	21.07 ± 10.18	42.38 ± 64.8	21.85 ± 7.81	20.37 ± 7.66	>0.05	>0.05
PDQ-5-D	11.93 ± 4.94	8.33 ± 5.42	7.60 ± 5.44	8.70 ± 3.81	6.35 ± 4.28	6.39 ± 4.57	>0.05	>0.05

*DLPFC* dorsolateral prefrontal cortex, *rTMS* repetitive transcranial magnetic stimulation, *W0* week 0, *W2* week 2, *W4* week 4, *P1* the interaction between time of measurement (W0/W2) and group (active/sham), *P2* the interaction between time of measurement (W2/W4) and group (active/sham).

### FC changes after rTMS treatment

#### DLPFC ROI-based FC

In the DLPFC active-sham group, we compared the whole-brain FC network with DLPFC (−32, 42, 32) seed between W0 and W2. This showed no significant change between the baseline and after active treatment (voxel-wise < 0.001, cluster-wise FWE-corrected, *P* > 0.05). In the DLPFC sham-active group, the whole-brain FC network with DLPFC seed was compared between W0 and W4, also showing no significant within-group changes between the baseline and after active treatment.

#### ACC ROI-based FC

In ACC active-sham group, we compared the whole-brain FC network with ACC (−4, 50, 4) seed between W0 and W2. No significant within-group change was observed between the baseline and after active treatment (voxel-wise < 0.001, cluster-wise FWE-corrected, *P* > 0.05). However, if not corrected by multiple comparisons, one significant cluster was present in the left hippocampus on comparison of the ACC ROI-based FC between the baseline and after active treatment, shown by the paired *t*-test (voxel-wise *p* < 0.001, cluster-wise FWE uncorrected *p* < 0.05, peak voxel MNI coordinates = [−24, −6, −24], cluster size = 20) (see Supplementary Fig. 2). However, no significant differences were seen on further analysis using the correlation between ACC-based FC changes and improvement in cognitive function. In the ACC sham-active group, we compared the whole-brain FC network with ACC seed between W0 and W4, finding that, whether corrected or not, the within-group changes did not differ between the baseline and after active treatment.

### Adverse effects

Adverse effects were mostly seen during and after active rTMS treatment. Of the 12 participants who experienced adverse effects, the effects were mild and temporary. These mainly included mild cephalgia, slight dizziness, nausea, sleepiness, transient earache, and pain at the site of stimulation. Only one participant in the ACC active-sham group experienced severe adverse effects (SAEs). This patient was on stable medication of quetiapine 500 mg and valproate 1000 mg per day and was excluded due to disease progression after 10 active rTMS sessions and two sham rTMS sessions. The patient was sent to the emergency room to have his stomach pumped on May 3, 2021, due to an overdose of about 20 pills (200 mg per pill) of quetiapine; he was discharged the following day and maintained on the same medication regimen until he went to the clinic on May 11, 2021. For several days after the overdose, the patient experienced mood instability, depression, and irritability with a score on the Chinese version of the Clinically Useful Depression Outcome Scale supplemented with DSM-5 Mixed subtype (CUDOS-M-C) of 19, which was much higher than the cut-off value of the standard of depression with mixed features [38]. This participant was stabilized and reviewed regularly after adjustment of his medication in the clinic. No SAEs were reported in the other participants.

## Discussion

It was found that rTMS targeting of the visual cortex functionally connected to the ACC led to a significant improvement in the accuracy of Symbol Check in the THINC-it tests, reflecting working memory, executive function, and attention. However, targeting of the VC functionally anti-correlated with the DLPFC did not have a significant effect, nor were any significant within-group changes in the FC between DLPFC/ACC and the whole brain observed between the baseline and after active treatment in any group. One participant in the ACC active-sham group experienced an SAE, specifically, disease progression and an overdose of medication after ten active and two sham rTMS sessions. Otherwise, active rTMS targeting of V1 was well-tolerated by the rest of the subjects with reports of SAEs. A series of case reports have shown a link between rTMS and the development of treatment-induced mania/hypomania [39, 40] and mixed states [41] in bipolar or unipolar depression, even in healthy subjects, when the left DLPFC was targeted. Mixed-state and manic/hypomanic episodes could result from rTMS in a similar manner to the use of antidepressants in BD; this was thought to be unrelated to medication as the medications used in some cases were only mood stabilizers [39]. However, as most of these studies investigated patients with complicated medications, including antidepressants, mood stabilizers, or atypical antipsychotics, it is difficult to clarify the reason for the treatment-emergent mood alteration [42]. In addition, in the present study, the participant had quarreled with his classmate the day before the suicide attempt, and this information was provided by the participant himself as the trigger for his overdose. Hence, the disease progression in this patient could be ascribed to a combination of psychosocial factors and an adverse reaction to rTMS. The current study appears to be the first to apply MRI-based neuro-navigated rTMS targeting of V1 in BD patients with cognitive impairment, and is the first to report the use of V1 as an effective and tolerant rTMS target for improving cognitive function in BD.

Previous data have demonstrated that some medications may provide potential benefits to cognitive impairment in euthymic BD patients (e.g., cholinesterase inhibitors [43], corticosteroid receptor antagonists [44], dopaminergic agonists [45], intranasal insulin [46], several antioxidants, and erythropoietin [47], amongst others). However, there are no specific recommended pharmacological treatments for cognitive impairment, as the reported findings are inconsistent. In contrast, non-pharmacological interventions, especially rTMS, have been shown to have promising efficacy in ameliorating cognitive impairment. A previous study recruited 52 participants with euthymic BD, who received ten sessions of active (50 5-s 10-Hz trains, 110% of the motor threshold) or sham rTMS within a double-blind, sham-controlled trial. The active rTMS group received 25,000 stimuli positioned over the left DLPFC. It was found that the rTMS enhanced cognitive function, including working memory and processing speed, without adverse effects [10]. A review of randomized controlled trials indicated that rTMS was more effective in BD patients during the remission phase, while its effect on the patients in the acute phases, especially depressive episodes, seemed to be limited [48]. There are several explanations for cognitive improvement after rTMS in BD. Wang et al. [49] found that 5-Hz rTMS administered daily for five consecutive days increased brain-derived neurotrophic factor (BDNF)-tyrosine receptor kinase B (TrkB) signaling in both the PFC and lymphocytes of rats, and decreased the resting motor threshold (RMT) and increased BDNF–TrkB signaling in human lymphocytes, suggesting a potential benefit to synaptic plasticity-related learning and memory mediated by the BDNF–TrkB pathway. Other studies have shown that the neural effects induced by rTMS were linked to increased synaptic plasticity [50] and changes in the excitability of the cerebral cortex [51]. Furthermore, rTMS may lead to neuroprotective effects through the modulation of oxidative injury, levels of BDNF, stress hormones, dopamine, and serotonin, BDNF, neuroinflammation, and the proliferation of hippocampal cells [52], while reducing both microglial activation and neuronal death in various brain regions and thus enhancing recovery of function [53].

Several researchers have recently focused on the visual cortex and studied its potential role in the development of the depression phenotype in depressive disorders. A recent study using fMRI-based neuro-navigated rTMS administered to 74 patients with depression showed that five days of active rTMS positioned over the VC was superior to sham stimulation in mitigating depressive symptoms, supporting the use of the VC as a stimulation target for effective rTMS treatment in MDD [24]. Here, we established a more stable and precise method of targeting the VC using fixed coordinates based on the patient’s MRI images, leading to an MRI-generated 3D curvilinear reconstruction of the brain. To avoid the interference of emotional effects on cognitive function, we recruited BD participants who were in the euthymic stage. Interestingly, two of the 18 participants in the ACC active-sham group subjectively reported unexpected improvements in their depressive symptoms after active rTMS. It is thus possible that cognitive enhancement in remitted BD patients produced by rTMS targeting of V1 may be partially attributed to the involvement of the VC and ACC in neural circuits related to anti-depressive effects [54, 55]. Recently, light therapy has been confirmed to have anti-depressive effects in both unipolar [56] and bipolar depression [57], involving a circuit linking the habenula, thalamus, amygdala, and VC [58]. The transmission of light signals from the retina has been shown to modulate emotional behavior, with the V1 being crucial for the transmission of visual information. Importantly, the V1 cortex was found to maintain a di-synaptic circuit linked to a subset of retinal neurons [59] and projecting to the superior colliculus [60], which regulated mood-related behavior via the amygdala [61], a critical region associated with emotional and cognitive processes in BD [62]. In addition, increased emotional responses in the right visual cortices of depressed subjects to specific facial stimuli were shown to predict good clinical outcomes of antidepressant medication, suggesting the crucial role of the visual cortex in depressive symptoms in depression [55].

Notably, there is also mounting evidence of the importance of the early VC (V1–V3) in working memory, through the maintenance of information associated with specific cognitive functions such as spatial orientation [63]. It has been reported that the volume, surface area, and thickness of V1 predicted performance in a visual working-memory task, suggesting that individuals with larger V1s were likely to have greater storage of visual working memory [20]. In contrast, inhibitory rTMS targeting the visual cortex could interrupt specific cognitive functions. A single-blind study was conducted on 34 healthy subjects treated with low-frequency (1 Hz), inhibitory rTMS over the early VC while controls were targeted over the vertex. The study showed that rTMS-inducing inhibition over the early VC reduced visual skill memory compared with control stimulation, suggesting that human perceptual memories associated with processing in the early VC were susceptible to rTMS even though the skills had been previously consolidated [64]. Thus, it is possible that, in the present study, TVCS may positively influence cognitive function through the regulation of early-visual cortical processing.

Additionally, neuroimaging studies have emphasized the association of ACC with affective disorders [65]. The dorsal ACC, in particular, is deemed to be important due to its close association with numerous cognitive functions, including executive function [29], detecting the possibility of error commission [66], monitoring conflict during competing responses [67], and reward monitoring [68], amongst others. A specific neural network has been found to be involved in cognitive tasks in patients with mood disorders, seen in significant activation or deactivation compared with healthy controls. This network includes the cingulate cortex (CC), especially the ACC [69, 70], which was found to display coordinated activation during cognitive control tasks and was thought to constitute part of the cognitive control network (CCN) [71]. In addition, the ACC is considered a crucial region participating in the executive attention network of the brain associated with functions such as conflict resolution and error detection [72, 73].

Furthermore, research on rodents has demonstrated that the ACC receives anatomical input from the VC [74] with rapid activation by simple visual stimuli [75]. It was found that direct projections from the VC to ACC microcircuits could be evoked by simple visual stimuli [76]. In addition, reduced FC was observed between the VC and the pre/subgenual ACC after high-frequency rTMS targeting over the V1, which was initially abnormally increased in MDD in resting-state fMRI [24]. Taken together, these findings suggest the potential mechanism by which rTMS targeting of VC-ACC FC led to the improvement of working memory, executive function, and attention in the present study. It is also possible that specific projections exist between the LGN, VC, and ACC that may modulate cognitive function, including depressive symptoms regulated by the retina-ventral LGN/intergeniculate leaflet (IGL)-lateral habenula (LHb) circuit, which has been regarded as a potential mechanism underlying the effects of light treatment on depression [58]. Further investigations are needed to verify our findings.

Although the FC between DLPFC/ACC and the whole brain did not show significant within-group changes, the results indicated the presence of stronger FC between the ACC and the left hippocampus after active rTMS treatment if the images were not corrected by multiple comparisons. Previous evidence indicated that active rTMS could induce significant changes not only in regions close to the stimulation site but also in more distal regions that are anatomically or functionally interconnected with the stimulation site [77–79]. For example, rTMS could modify the resting-state FC between parietal regions and the hippocampus, which was found to be strong at the baseline. Nevertheless, such modulation between the ACC and visual cortex was not apparent in the present study, possibly due to the small sample size. Consistent with the findings of the present study, an earlier study also suggested that the effects of rTMS on resting-state FC were most apparent in areas outside the network of the stimulated region, indicating that the effects of rTMS tend to diffuse across networks [80]. Furthermore, the hippocampus is crucial for memory modulation in BD [81]. The enhancement of the FC between the left hippocampus and the ACC may partially account for improved cognitive function. These results may also suggest that the effects of rTMS do not necessarily correspond with the area of stimulation and may produce effects in more distal regions [80].

There were some limitations to the present study. First, the experimental design using the time to acquire the second fMRI data after active treatment did not allow a comparison of the difference between the effects on FC between active and sham rTMS. Second, the stimulating coordinate in the V1 was calculated based on the group level, instead of individually targeting regions functionally relevant to the DLPFC or ACC, and the coordinates of the DLPFC and ACC were selected according to two previous studies instead of our own dataset of the subjects enrolled in the study. Third, there were deficiencies in data in terms of follow-up with no rTMS sessions; further studies are required to verify long-term effects after the completion of the rTMS treatment. Fourth, the sample size was fairly small, and the numbers of participants in four groups were unsymmetrical. Fifth, there was a lack of control groups. Sixth, medications that may have influenced the cognitive function of the participants in each group were not the same. Finally, the cognitive test inevitably confronts the challenge of practice effects, and longer follow-up times are needed to avoid this problem in future research.

## Conclusion

In summary, neuro-navigated rTMS targeting the V1 functionally connected to the ACC was found to improve attention, working memory, and executive function in remitted BD patients with good tolerance. It is thus suggested that the V1 is a potentially effective stimulation target for neuro-navigated rTMS for the enhancement of cognitive function and may form a significant part of the pathophysiological mechanism underlying cognitive impairment in BD. Additionally, TVCS provides a novel neural-stimulating mode.

## Supplementary information

Supplementary Table and Figure

## Data Availability

The datasets used in the present study are not publicly available to protect the privacy of participants but are available from the corresponding author upon reasonable request.
