# Supplementary material for: Targeted visual cortex stimulation (TVCS): a novel neuro-navigated repetitive transcranial magnetic stimulation mode for improving cognitive function in bipolar disorder
Source: Transl Psychiatry. 2023 Jun 8;13:193. doi: 10.1038/s41398-023-02498-z (PMC10250373; doi:10.1038/s41398-023-02498-z)
Supplement: Supplementary file 1 — Supplementary Table and Figure [file 41398_2023_2498_MOESM1_ESM.docx]

Supplementary figure

Figure 1. Two coordinates in V1 chosen as the TMS targets in remitted BD participants

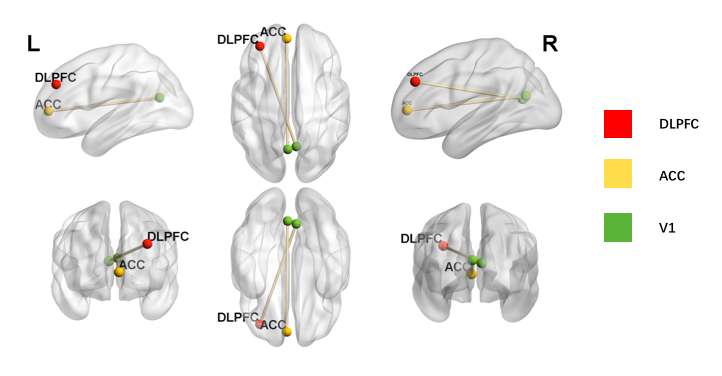


S2(-3, -66,18)

S1 (6, -63,15)

Regions of interests (ROIs) in the DLPFC and ACC were based on previously reported coordinates from prior studies involved in bipolar disorder. Red color refers to ROIs in the DLPFC (-32, 42, 32), yellow color refers to ROIs in the ACC (-4, 50, 4), green color refers to V1 stimulation sites. We defined DLPFC and ACC seeds by 9 mm spheres centered on the coordinates (-32, 42, 32) (-4, 50, 4) and used these two seeds to construct functional connectivity maps. V1 stimulation site (6, -63,15) showed significant anticorrelations with the DLPFC (voxelwise p< 0.001, clusterwise FWE corrected p<0.05, r coefficient was -0.179), V1 stimulation site (-3, -66,18) showed significant correlations with the ACC (voxelwise p< 0.001, clusterwise FWE corrected p<0.05, r coefficient was 0.269). DLPFC, dorsolateral prefrontal cortex; ACC, anterior cingulate cortex; V1, primary visual cortex.

Figure 2. Within group comparison of ACC based whole-brain FC network between W0 and W2.


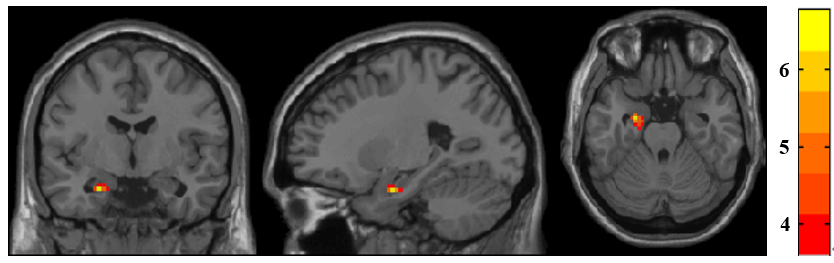


One significant cluster was found in the left hippocampus (voxel-wise p< 0.001, cluster-wise FWE uncorrected p<0.05, peak voxel MNI coordinates = [-24, -6, -24], cluster size = 20). Colorbar shows the F value which indicates the degree of the difference.

Supplementary table

Table1. Description of Cognitive Tasks in Conventional and THINC-it Tests

|  | Conventional neuropsychological tests | [Scoring](javascript:;) [method](javascript:;) | Cognitive domain |  |
| --- | --- | --- | --- | --- |
| THINC-it |  |  |  | Direction |
|  |  |  |  |  |
| Spotter | IDN | Mean of the log-transformed reaction time (seconds) | Attention | negative |
| Symbol Check | OBK | Mean of the log-transformed reaction time (seconds);  accuracy of trials (arcsine square root of proportion of correct responses) | Working memory, executive function, and attention. | Negative;  positive |
| Codebreaker | DSST | Total number correct | Executive functions, processing  speed, and attention, | Positive |
| Trails | TMT-B | Time to complete (seconds) | Executive function | Negative |
| PDQ-5-D | PDQ-D | Sum of items | Cognitive function of capturing  subjective perception | Negative |

THINC-it, THINC-integrated tool; IDN, Identification Task; OBK, One Back Test; DSST, Digit Symbol Substitution Test; TMT-B, Trail Making Test-Part B; PDQ-5-D, Perceived Deficits Questionnaire for Depression-5-item; PDQ-D, Perceived Deficits Questionnaire for Depression;

A “positive” direction means the scores of the test are positively correlated with corresponding cognitive function; a “negative” direction means the scores of the test are negatively correlated with corresponding cognitive function.

Supplementary table

Table 2. Statistical analysis of YMRS and HDRS score in four groups

|  | DLPFC | | | | | | | | | ACC | | | | | | | | | Analysis  *F/χ^2^* | *p*-value |
| --- | --- | --- | --- | --- | --- | --- | --- | --- | --- | --- | --- | --- | --- | --- | --- | --- | --- | --- | --- | --- |
|  | A1 (n=15) | | | A2 (n=23) | | | | |  | B1 (n=18) | | | | B2 (n=17） | | | | |  |  |
|  | W0 | W2 | W4 | W0 | | W2 | W4 | | | W0 | | W2 | W4 | W0 | | W2 | W4 | |  |  |
| YMRS score | 1.13±  1.64 | 0.73±  1.03 | 0.60±  0.99 | 1.43±  2.25 | 0.79±  1.62 | | | 0.52±  1.31 | | 2.06±  2.51 | 1.33±  1.68 | | 1.22±  2.02 | 2.00±  1.97 | | 2.18±  3.94 | | 0.94±  1.09 | 1.315 | 0.277 |
| HDRS score | 4.40±  2.32 | 3.80±  2.48 | 3.27±  2.34 | 3.78±  2.54 | 2.91±  2.37 | | | 2.39±  2.64 | | 4.11±  2.52 | 2.94±  1.89 | | 2.28±  2.32 | 5.59±  1.62 | 4.06±  2.38 | | | 3.29±  2.25 | 1.590 | 0.200 |

DLPFC, dorsolateral prefrontal cortex; ACC, anterior cingulate cortex; YMRS, Young Manic Rating Scale; HDRS, Hamilton Depression Rating Scale; W0, week 0; W2, week 2; W4, week 4; A1, DLPFC active-sham rTMS Group; A2, DLPFC sham-active rTMS Group; B1, ACC active-sham rTMS Group; B2, ACC sham-active rTMS Group.

The p-value is 4 groups (A1, A2, B1, B2) × 3 time (week 0, week 2, week 4) repeated-measures ANOVA result, p＜0.05

Supplementary table

Table 3. Interaction between time of measurement and stimulation target (ACC/DLPFC) on six THINC-it cognitive function scores in participants with active-sham stimulation and sham- active stimulation

|  | Active-sham | | | | | | | | Sham-active | | | | | | | | P1 | P2 |
| --- | --- | --- | --- | --- | --- | --- | --- | --- | --- | --- | --- | --- | --- | --- | --- | --- | --- | --- |
|  | A1 (n=15) | | | B1 (n=18) | | | |  | A2 (n=23) | | | | B2 (n=17） | | | |  |  |
|  | W0 | W2 | W4 | W0 | W2 | W4 | | | W0 | | W2 | W4 | W0 | W2 | W4 | |  |  |
| Spotter | -0.20±  0.15 | -0.27±  0.12 | -0.30±  0.11 | -0.26±  0.13 | -0.30±  0.11 | | -0.31±  0.10 | | -0.22±  0.13 | -0.25±  0.11 | | -0.26±  0.11 | -0.20±  0.15 | -0.29±  0.08 | | -0.31±  0.08 | >0.05 | >0.05 |
| Symbol Check (Time) | 0.01±  0.11 | -0.02±  0.09 | -0.05±  0.10 | 0.03±  0.11 | -0.04±  0.09 | | -0.08±  0.08 | | 0.05±  0.10 | 0.02±  0.09 | | -0.04±  0.08 | 0.06±  0.11 | -0.03±  0.07 | | -0.07±  0.08 | >0.05 | >0.05 |
| Symbol Check (Accuracy) | 0.54±  0.31 | 0.71±  0.26 | 0.74±  0.29 | 0.51±  0.25 | 0.67±  0.3 | | 0.71±  0.28 | | 0.43±  0.27 | 0.53±  0.29 | | 0.66±  0.26 | 0.56±  0.29 | 0.6±  0.28 | | 0.75±  0.24 | >0.05 | >0.05 |
| Code breaker | 49.8±  17.17 | 56.8±  21.51 | 59.67±  21.75 | 48.44±  16.23 | 57.00±  23.66 | | 59.72±21.68 | | 42.91±  16.58 | 53.04±12.26 | | 56.57±14.73 | 54.88±17.92 | 61.76±  16.63 | | 65.94±  12.66 | >0.05 | >0.05 |
| Trails | 27.51±14.52 | 22.65±9.00 | 21.07±  10.18 | 77.51±  200.46 | 38.28±59.06 | | 34.11±64.99 | | 42.38±  64.80 | 21.85±7.81 | | 20.37±7.66 | 29.63±20.54 | 22.12±  11.21 | | 19.17±  10.13 | >0.05 | >0.05 |
| PDQ-5-D | 11.93±4.94 | 8.33±  5.42 | 7.60±  5.44 | 11.17±  3.78 | 8.78±  3.19 | | 7.33±  2.99 | | 8.70±  3.81 | 6.35±  4.28 | | 6.39±  4.57 | 10.76±5.23 | 9.88±  4.85 | | 8.94±  4.42 | >0.05 | >0.05 |

A1, DLPFC active-sham rTMS Group; A2, DLPFC sham-active rTMS Group; B1, ACC active-sham rTMS Group; B2, ACC sham-active rTMS Group; W0, week 0; W2, week 2; W4, week 4; P1, the interaction between time of measurement (W0/W2) and group (ACC/DLPFC); P2, the interaction between time of measurement (W2/W4) and group (ACC/DLPFC)
